# Characterisation of the protective immune response following subcutaneous vaccination of susceptible mice against *Trichuris muris*

**DOI:** 10.1016/j.ijpara.2009.11.008

**Published:** 2010-05

**Authors:** Helen Dixon, Matthew C. Little, Kathryn J. Else

**Affiliations:** Faculty of Life Sciences, University of Manchester, Michael Smith Building, Oxford Road, Manchester M13 9PT, UK

**Keywords:** Vaccination, Gastrointestinal nematode, *Trichuris*, Th2 cells, Antibody

## Abstract

*Trichuris muris* is a laboratory model for the human whipworm *Trichuris trichiura* which infects approximately 1 billion people in tropical and sub-tropical countries. The development of a vaccine would control trichuriasis by promoting the acquisition of immunity during childhood, thereby reducing faecal egg output by the community into their environment. Resistance to *T. muris*, defined as expulsion of the parasite prior to patency, requires the development of a T helper 2 (Th2) response during a primary infection. To our knowledge this is the first study to describe the protective immune response in the peripheral lymph nodes (PLN), mesenteric lymph nodes (MLN) and colonic mucosa following s.c. vaccination against *T. muris*. Susceptible AKR mice were either vaccinated with *T. muris* excretory–secretory product (ES) in incomplete Freund’s adjuvant (IFA) (ES/IFA) or injected with PBS in IFA (PBS/IFA) and for protection experiments were infected with embryonated infective *T. muris* eggs 10 days later. The ES/IFA vaccine induced the proliferation of PLN cells and their production of Th2 cytokines and the Th1-associated cytokine IFN-γ. Following a challenge infection, the ES/IFA vaccination offered susceptible mice complete protection. While MLN-derived IFN-γ was produced by infected mice following either ES/IFA vaccination or PBS/IFA, the protection of susceptible mice by ES/IFA was characterised by the production of MLN-derived Th2 cytokines. Goblet cell hyperplasia and the influx and alternative activation of macrophages were observed locally in the gut post-challenge infection. The rate of epithelial turnover did not appear to be increased by vaccination, suggesting that there are differences in the mechanisms of expulsion between ‘natural resistance’ and ‘vaccinated resistance’. High levels of serum IgG1 and cell-bound IgG1 in the colon of mice protected by the ES/IFA vaccine suggest that antibody may be involved in vaccination-induced worm expulsion.

## Graphical abstract

**Figure:**
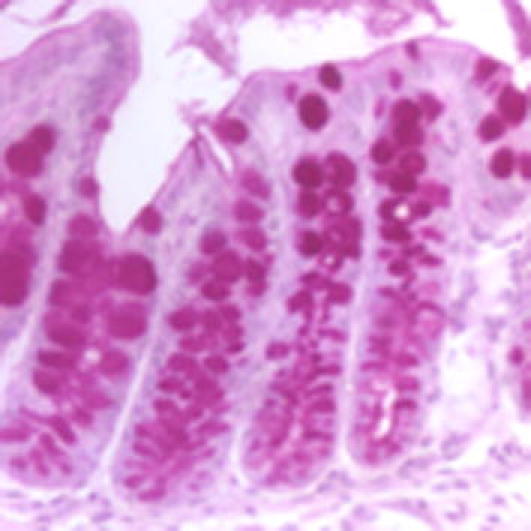

## 1 Introduction

*Trichuris trichiura*, the human whipworm, infects approximately 1 billion people in tropical and sub-tropical countries (Chan, 1997). Anthelminthic drugs effectively clear gut-dwelling nematode parasites but are not a practical solution to trichuriasis since infection is soon re-established in the human population from their environment. Rather, long-term immunological intervention strategies are required to combat the disease. *Trichuris muris* infection of mice may serve as a useful model in which to investigate the design of a human vaccine since strong type 2 immune responses are associated with host resistance to both *T. trichiura* (Jackson et al., 2004) and *T. muris* (Else and Grencis, 1991). The range of protective immunity mounted against *T. muris* in the mouse model of infection varies depending on the background genetics of the inbred strain of mouse (Else et al., 1990). Some strains of mouse, such as BALB/c, are resistant to *T. muris* and quickly expel the parasite, whereas others such as AKR are susceptible, allowing the development of a chronic infection of the cecum and proximal colon characterised by the presence of fecund adult parasites.

Following a primary *T. muris* infection, it is now understood that resistance requires the development of a type 2 response typified by the release of mesenteric lymph node (MLN)-derived Th2 cytokines leading to high levels of IgG1 in the serum. Susceptibility occurs following the development of an inappropriate Th1 response, which involves the release of IL-12 and IFN-γ by MLN cells, leading to elevated serum IgG2a levels (Else et al., 1993). While the exact expulsion mechanism during primary infection is yet to be elucidated, epithelial turnover, (Cliffe et al., 2005) goblet cells (Artis et al., 2004) and macrophages (Deschoolmeester et al., 2003; Little et al., 2005) have all been implicated. In isolation, neither mast cells, eosinophils, antibodies nor antibody-dependent cellular cytotoxicity (ADCC) are essential for the resolution of primary infections (Else and Grencis, 1996; Betts and Else, 1999; Betts et al., 2000). In contrast, little is known about the expulsion mechanisms in a secondary (challenge) infection.

In previous studies, resistant NIH (Wakelin and Selby, 1973; Jenkins and Wakelin, 1977, 1983), poorly resistant C57BL/10 and susceptible B10.BR (Else and Wakelin, 1990; Robinson et al., 1995) strains of mouse were successfully protected by a s.c. vaccination consisting of *T. muris* adult excretory–secretory product (ES) in either FCA or incomplete Freund’s adjuvant (IFA). However, neither the immune response to vaccination nor the mechanisms underlying worm expulsion, following a challenge infection, were studied in detail. This study investigates the immune response to s.c. vaccination with adult *T. muris* ES in IFA in the highly susceptible AKR strain of mouse. Furthermore, this study examines the potential effector mechanisms involved in the clearance of a challenge infection.

## 2 Materials and methods

### 2.1 Animals

Specific-pathogen-free male AKR and BALB/c mice (Harlan, Bicester, UK) were maintained in micro-isolator cages in the animal facility at the University of Manchester. Mice were vaccinated and infected at 6–8 weeks old. They were fed autoclaved food and water and all manual procedures were carried out under sterile-filtered laminar air flow conditions.

All procedures carried out on animals were performed under a Home Office project license and complied at all times with UK laws and regulations. All animal work was performed under the regulations of the Home Office Scientific Procedures Act (1986).

### 2.2 Parasite

The *T. muris* parasite was maintained and the mice were infected with eggs by oral gavage (Wakelin, 1967). Worm burdens were assessed (Else et al., 1990) and worm ES antigens were prepared (Bancroft et al., 1998). Briefly, live adult worms were cultured for 4 h at 37 °C and the ES antigens were prepared for the later in vitro re-stimulation of MLN cells. The culture medium was replenished and the worms were incubated overnight at 37 °C. This yielded ES antigens for the preparation of the vaccine and for use in antibody ELISAs and Western blots.

### 2.3 Vaccination

Sterile-filtered ES antigens were emulsified with an equal volume of IFA (Sigma–Aldrich, Dorset, UK) to achieve a vaccine dose of 100 μg ES in 100 μl (1 mg/ml ES/IFA). Accordingly, control mice were injected with 100 μl PBS/IFA. In the standard protocol, vaccinations or control injections were administered s.c. in one abdominal site and an infection was given 10 days later. Alternatively, mice were vaccinated with a total vaccine dose of 100 μg ES in 100 μl ES/IFA or injected with PBS/IFA split between several s.c. sites at either 12, 9, 6 or 3 days prior to autopsy but were not subsequently infected. The latter approach was taken in order to investigate the proliferation of peripheral lymph node (PLN) cells and their release of cytokines.

### 2.4 In vitro re-stimulation of MLN and PLN cells

The MLN and the axillary, brachial and inguinal PLN were removed at autopsy and dissociated in RPMI-1640 media supplemented with 10% FCS, 2 mM l-glutamine, 100 U/ml of penicillin, 100 μg/ml streptomycin (all Invitrogen Ltd., Paisley, UK). MLN or PLN cells (5 × 10^6^ per well in 48-well plates) were re-stimulated in vitro with *T. muris* ES antigens (50 μg/ml) at 37 °C. The supernatants were collected after 48 h and stored at −20 °C until cytokine analysis was performed. Alternatively, the lymph node cells (1 × 10^6^ per well in 96-well plates) were re-stimulated in vitro with *T. muris* ES antigens (50 μg/ml) at 37 °C for 72 h prior to the measurement of proliferation by the MTT [3-(4,5-dimethylthiazol-2-yl)-2,5-diphenyl tetrazolium bromide] assay.

### 2.5 Cytokine analysis

Sandwich ELISAs were used to determine the concentrations of IL-5, IL-9, IL-12, IL-13 and IFN-γ in the supernatants recovered from in vitro-cultured MLN and PLN cells, as described previously (Taylor et al., 2000). The IL-13 ELISA was performed using the Quantikine M Mouse IL-13 Colorimetric Sandwich ELISA kit (R&D Systems, Abingdon, UK).

### 2.6 MTT eluted stain assay

Cell proliferation was measured using the MTT eluted stain assay (Mosmann, 1983). Briefly, 50 μg/ml MTT (Sigma–Aldrich) was added to each well and the plate cultured at 37 °C for 60 min. Following this, 100 μl of acidified isopropanol was added to each well and the wells were mixed by pipetting. The plate was read at 560 nm on a MRX II microplate reader (Dynex Technologies Ltd., Worthing, West Sussex, UK) with the reference filter set at 630 nm.

### 2.7 Histology

At autopsy, colonic tissue samples were taken from the site immediately distal to the cecum. Samples were fixed at room temperature for 4 h in Carnoy’s fixative, or overnight in 10% neutral-buffered formalin, prior to processing and embedding in paraffin wax. Serial sections, 5 μm thick, were de-waxed in citroclear and rehydrated prior to staining with either H&E, toluidine blue or Schiff’s reagent which stain eosinophils, mast cells or goblet cells, respectively. The number of each cell type in the colonic epithelium was counted and expressed per 20 crypt units.

### 2.8 Crypt length

Photographs of colonic sections for crypt length measurement were taken at 100× magnification on a Nikon Eclipse E400 microscope using SPOT Advanced version 3.5.5 software. Twenty crypt lengths were measured per mouse using Image-Pro Plus version 4.5.1.22 image-analysis software and the average crypt length per mouse was calculated.

### 2.9 Immunohistochemistry

Colorimetric immunohistochemistry, employing standard immunoperoxidase techniques (Little et al., 2005), was carried out using one of the following biotinylated rat, anti-mouse monoclonal antibodies (mAbs): anti-IgG1 (5 μg/ml; BD Biosciences, Oxford, UK), anti-IgA (5 μg/ml; BD Biosciences), anti-CD4 (7.5 μg/ml; BD Biosciences), anti-B220 (10 μg/ml; BD Biosciences), anti-F4/80 (7.5 μg/ml; Serotec, Oxford, UK). Alternatively, staining was achieved using either purified rabbit anti-mouse iNOS (5 μg/ml; BD Biosciences) or rat anti-mouse FIZZ1 (5 μg/ml; R&D Systems, Abingdon, UK) followed by a biotinylated secondary antibody (Ab) (Chemicon International, California, USA). The sections were incubated in parallel with the appropriate biotinylated or purified isotype control Abs, as applicable (BD Biosciences). The blue nuclear dye Hematoxylin QS (Vector Laboratories Ltd., Peterborough, UK), was used as a counter-stain. The number of positively stained cells in 20 crypts was counted at high magnification using a Nikon Eclipse E400 microscope. Digital photographs were taken using SPOT Advanced version 3.5.5 software.

In brief, for dual immunofluorescence staining, the sections were incubated for 1 h with either biotinylated anti-IgG1 or anti-IgA (BD Biosciences) followed by avidin-conjugated Texas Red (30 μg/ml; Vector Laboratories Ltd.) for 30 min. Avidin and biotin binding sites were then saturated using an avidin/biotin blocking kit (Vector Laboratories Ltd.). The sections were then incubated with either biotinylated anti-B220 or anti-F4/80 (BD Biosciences) for 1 h, followed by avidin-conjugated FITC for 30 min (30 μg/ml; Vector Laboratories Ltd.). The sections were mounted in Vectorshield mounting medium containing the blue nuclear counter-stain DAPI (Vector Laboratories Ltd.). Digital images were captured using an Olympus Bx-1 fluorescence microscope fitted with a Coolsnap camera and Metavue software.

### 2.10 Parasite-specific antibody detection in the serum

Blood was taken immediately postmortem by cardiac puncture and left at room temperature to clot. The serum was collected from each sample, then aliquoted and stored at −20 °C prior to the analysis of parasite-specific IgG1 and IgG2a levels by ELISA as previously described (Else et al., 1993).

### 2.11 Statistical analyses

The data presented here are from one of two experiments. All groups contained five animals at each time-point unless otherwise stated. Significant differences between multiple groups (*P* < 0.05) were determined by the Kruskall–Wallis non-parametric ANOVA with the Mann–Whitney *U* test modified by the Bonferroni correction as post-test. Significant differences between two groups were determined by the Mann–Whitney *U* test. Statistical analyses were carried out using GraphPad Prism software, version 3.02.

## 3 Results

### 3.1 ES/IFA vaccine protects susceptible AKR mice from *T. muris*

**Fig. 1:**
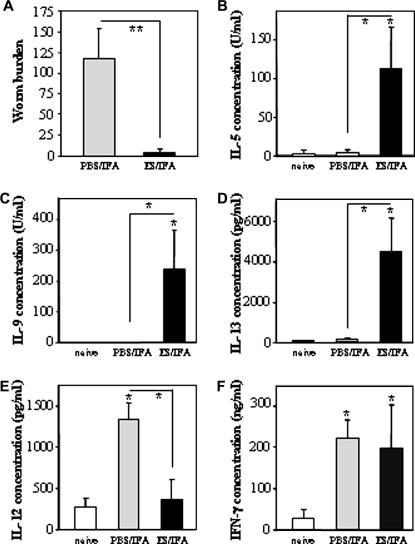
Worm burden and mesenteric lymph node cell (MLNC) cytokine profile at day 21 p.i. (A) Worm burdens and (B–F) cytokine analyses of MLNC supernatants on day 21 p.i. with *Trichuris muris* in naive, PBS/incomplete Freund’s adjuvant (IFA)-injected and excretory–secretory product (ES)/IFA-vaccinated AKR mice. Mice in the PBS/IFA-injected and ES/IFA-vaccinated groups were infected with approximately 150 infective embryonated *T. muris* eggs on day 0. MLNC supernatants were analysed for the presence of (B) IL-5, (C) IL-9, (D) IL-13, (E) IL-12p40 and (F) IFN-γ. Results are shown as means ± SD (*n *= 5 per group). * above error bar indicates cytokine levels significantly above naive levels (*P *< 0.05). A significant difference between infected groups is indicated by * with a line (*P *< 0.05) or ** with a line (*P *< 0.01). The results presented are from one of two experiments.

The ES/IFA vaccine induced a mean protection rate of 97% by day 21 p.i. (Fig. 1A). This consisted of 100% expulsion in two mice and 99%, 95% and 91% expulsion in the other three mice. By day 35 p.i. the ES/IFA vaccine had induced 100% expulsion in all five AKR mice (data not shown).

### 3.2 Protection is associated with the release of Th2 cytokines by MLN cells following vaccination and a subsequent challenge infection

The MLN cells from ES/IFA-vaccinated mice at 21 days p.i. released high levels of the Th2 cytokines IL-5, IL-9 and IL-13 upon their re-stimulation in vitro with worm ES antigens (Fig. 1B–D). In contrast, the MLN cells from both PBS/IFA-injected control mice at 21 days p.i. and untreated naive mice produced these cytokines only at low levels (Fig. 1B–D). Post-infection, MLN cells from PBS/IFA-injected control mice, but not ES/IFA-vaccinated mice, produced higher levels of the Th1 cytokine IL-12p40 compared with MLN cells from untreated naive mice (Fig. 1E). However, MLN cells from both the ES/IFA-vaccinated and the PBS/IFA-injected control mice released higher levels of the Th1 cytokine IFN-γ p.i. compared with those from untreated naive mice (Fig. 1F).

### 3.3 Vaccination (without a subsequent challenge infection) induces the proliferation of, and release of Th2 cytokines by, PLN cells

**Fig. 2:**
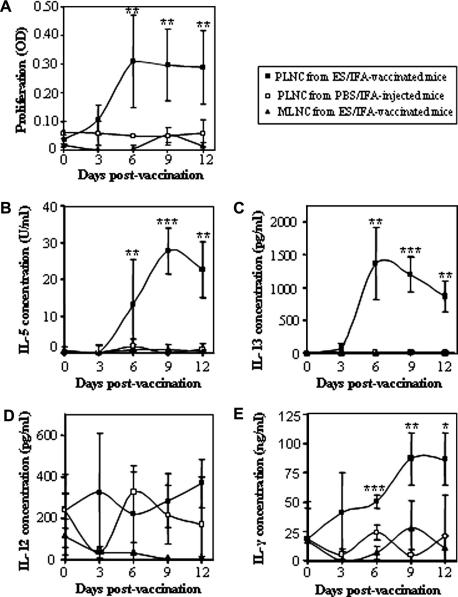
Cell proliferation and cytokine production profiles of peripheral lymph node cells (PLNC) and mesenteric lymph node cells (MLNC) following excretory–secretory product (ES)/incomplete Freund’s adjuvant (IFA) vaccination or PBS/IFA injection. (A) Cell proliferation as measured by MTT [3-(4,5-dimethylthiazol-2-yl)-2,5-diphenyl tetrazolium bromide] eluted stain assay and (B–E) cytokine analyses of in vitro ES-re-stimulated MLNC (triangles) and PLNC (squares) harvested from PBS/IFA-injected (open symbols) and ES/IFA-vaccinated (black symbols) AKR mice. Mice were vaccinated 12, 9, 6 and 3 days prior to autopsy. Results are shown as means ± SD. For all time-points *n *= 6 per group except in the MTT assay where on day 6 *n *= 2 for PLNC from PBS/IFA-injected AKR mice. Significant differences between the three groups are indicated by ∗*P *< 0.05, ∗∗*P *< 0.01, ∗∗∗*P *< 0.001. The results presented are from one of two experiments.

From 6 days post-vaccination, ES antigens induced the proliferation in vitro of PLN cells from ES/IFA-vaccinated unchallenged mice. This was not observed, at any time-point, in PLN cells from PBS/IFA-injected unchallenged control mice nor in MLN cells from ES/IFA-vaccinated unchallenged mice (Fig. 2A).

Accordingly, PLN cells (from the ES/IFA-vaccinated group of mice only), but not MLN cells, produced the Th2 cytokines IL-5 and IL-13 in response to stimulation in vitro by ES antigens (Fig. 2B and C). Neither vaccination with ES/IFA, nor control treatment with PBS/IFA, resulted in the elevation of IL-12p40 release (Fig. 2D) by PLN- or MLN-derived cultured cells. However, another Th1 cytokine, IFN-γ, was produced by PLN (but not MLN) cells taken from ES/IFA-vaccinated mice but not from PBS/IFA-injected control mice (Fig. 2E).

### 3.4 Crypt hyperplasia is observed in ES/IFA-vaccinated and PBS/IFA-injected mice following a challenge infection

**Fig. 3:**
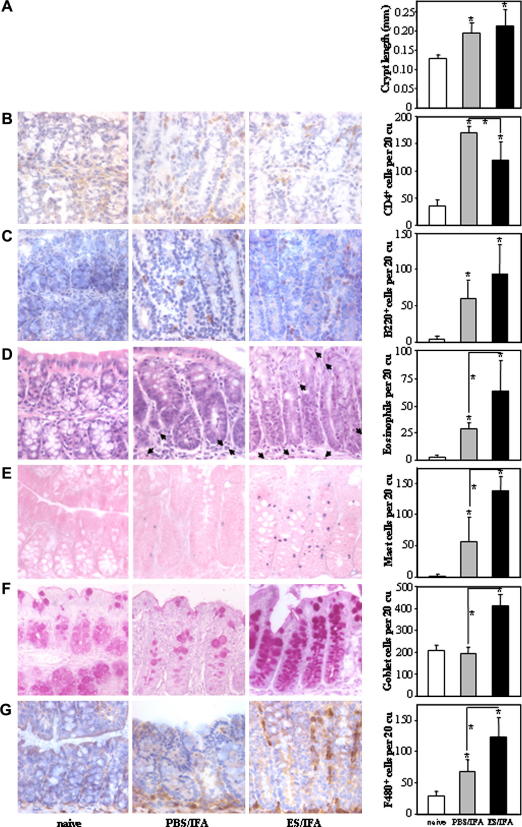
Colonic crypt length and cell infiltration. Representative photographs and numbers of (A) colonic crypt lengths and (B) CD4^+^ (C) B220^+^ (D) eosinophil (E) mast (F) goblet and (G) F4/80^+^ cells in the colonic mucosa of naive and infected PBS/incomplete Freund’s adjuvant (IFA)-injected and excretory–secretory product (ES)/IFA-vaccinated AKR mice on day 21 p.i. CD4^+^, B220^+^ and F4/80^+^ cells appear brown in immunohistochemical (IHC) stained sections. Eosinophils have a blue nucleus and pink cytoplasm in H&E stained sections and examples are marked by an arrow. Mast cells appear blue and granular in toluidine blue while goblet cells appear pink-purple in Schiff’s stained sections. All photographs shown were taken at 400× original magnification. Graphed results are shown as mean cell numbers per 20 crypt units (cu) ± SD (*n *= 5 per group). * above the error bar indicates that cell numbers are significantly above naive levels (*P *< 0.05). A significant difference between infected groups is indicated by * with a line (*P *< 0.05). The results presented are from one of two experiments.

The length of colonic crypts was measured in order to serve as an index of crypt hyperplasia. Prior to a challenge infection, there was no difference in the average length of colonic crypts between naive mice, ES/IFA-vaccinated mice and PBS/IFA-injected mice (10 days post-treatment, not shown). However, following a challenge infection, both the ES/IFA-vaccinated mice and the PBS/IFA-injected mice exhibited crypt hyperplasia (Fig. 3A).

### 3.5 A description of the cellular infiltrate following vaccination and subsequent challenge infection

The number of colonic CD4^+^ T cells was higher in both PBS/IFA-injected challenged and ES/IFA-vaccinated challenged mice than in untreated uninfected naive mice (Fig. 3B). There were significantly more CD4^+^ cells in the chronically infected PBS/IFA-injected control group compared with the vaccinated protected group (Fig. 3B). Compared with untreated, uninfected naive mice, there were more colonic B220^+^ B cells (Fig. 3C), eosinophils (Fig. 3D) and mast cells (Fig. 3E) in both PBS/IFA-injected challenged and ES/IFA-vaccinated challenged mice. The recruitment of B220^+^ B cells (Fig. 3C), eosinophils (Fig. 3D) and mast cells (Fig. 3E) to the colon was higher in protected ES/IFA-vaccinated mice than in chronically infected PBS/IFA-injected control mice. Compared with untreated uninfected naive mice, there were more colonic goblet cells in ES/IFA-vaccinated challenged mice but not in PBS/IFA-injected challenged control mice (Fig. 3F). Thus, the colons of protected ES/IFA-vaccinated mice contained more goblet cells than those of chronically infected PBS/IFA-injected control mice. There were more colonic F4/80^+^ macrophages in both the ES/IFA-vaccinated and PBS/IFA-injected challenged groups compared with the untreated naive group (Fig. 3G). The colons of protected ES/IFA-vaccinated mice contained more F4/80^+^ macrophages than the colons of chronically infected PBS/IFA-injected mice (Fig. 3G). Furthermore, F4/80^+^ macrophages were found mainly at the base of the crypts in PBS/IFA-injected mice but were found both at the base and along the length of the crypts in ES/IFA-vaccinated mice (Fig 3G). Each of the aforementioned types of cell was also quantified before the challenge infection (10 days post-treatment). In this case, neither the ES/IFA vaccine nor the PBS/IFA injection affected the number of these cells in the colon compared with untreated naive controls (data not shown).

### 3.6 ES/IFA vaccination induces alternatively activated macrophages

**Fig. 4:**
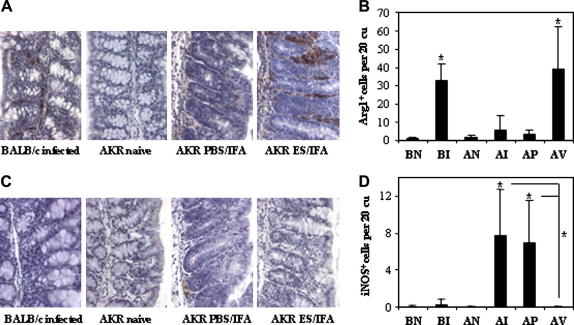
Excretory–secretory product (ES)/incomplete Freund’s adjuvant (IFA) vaccination induces alternatively activated macrophages. (A and C) Representative photographs and (B and D) numbers of (A and B) Arg1^+^ and (C and D) iNOS^+^ cells in the colonic mucosa of: naive BALB/c (BN); naive AKR (AN); infected BALB/c (BI); infected AKR (AI); PBSIFA-injected and infected AKR (AP); ES/IFA-vaccinated and infected AKR (AV) mice on day 21 p.i. Arg1^+^ and iNOS^+^ cells appear brown in immunohistochemical (IHC) stained sections. All photographs were taken at 400× original magnification. Graphed results are shown as mean cell numbers per 20 crypt units (cu) ± SD (*n *= 5 per group). (B and D) * above the error bar indicates that cell numbers are significantly above naive levels (*P *< 0.05). (D) * with a line indicates a significant difference (*P *< 0.05) between the infected PBS/IFA-injected and ES/IFA-vaccinated groups and between the infected non-vaccinated AKR and ES/IFA-vaccinated groups. The results presented are from one of two experiments.

The colonic macrophages were further characterised 21 days p.i. for iNOS and Arg1 expression, markers of classical and alternative activation, respectively (Lyons et al., 1992; Loke et al., 2002). These cells were quantified not only in our AKR mouse model of vaccination but also in naturally resistant BALB/c mice. Arg1^+^ and iNOS^+^ cells in gut tissue are quantified in Fig. 4B and D with images from pertinent groups shown as examples of staining in Fig. 4A and C. There were few colonic Arg1^+^ cells in untreated naive mice of either strain. This was also observed post-infection in PBS/IFA-injected control AKR mice (Fig. 4A and B). However, there were significantly more colonic Arg1^+^ cells in the ES/IFA-vaccinated AKR mice post-challenge with *T. muris* infection. This mirrored the increase in Arg1^+^ cells in resistant BALB/c mice as part of their natural immune response to the parasite (Fig. 4A and B). There were few colonic iNOS^+^ cells in untreated naive mice of either strain (Fig. 4C and D). Neither the natural immune response in resistant BALB/c mice, nor the successful immune response in vaccinated AKR mice to a challenge infection involved an increase in the number of colonic iNOS^+^ cells (Fig. 4C and D). In contrast, such an increase was observed both during the unsuccessful natural immune response of AKR mice to *T. muris* and in PBS/IFA-injected control AKR mice upon a challenge infection (Fig. 4C and D).

### 3.7 Serum antibody responses following ES/IFA vaccination

**Fig. 5:**
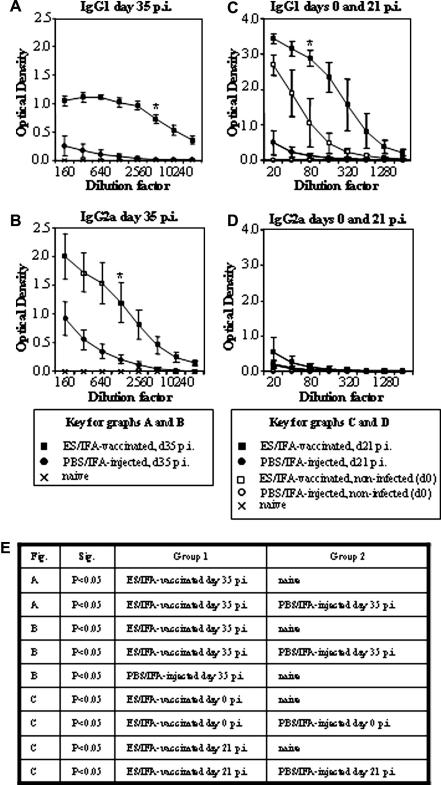
Serum antibody responses following ES/IFA vaccination. Sera from naive and *Trichuris muris*-infected PBS/incomplete Freund’s adjuvant (IFA)-injected and excretory–secretory product (ES)/IFA-vaccinated AKR mice were assayed by ELISA for parasite-specific (A and C) IgG1 and (B and D) IgG2a at (A and B) day 35 p.i. and (C) IgG1 and (D) IgG2a levels are compared between day 0 and day 21 p.i. Results are shown as means ± SD (*n *= 5 per group). Naive levels are depicted by a cross, PBS/IFA-injected levels by a circle and ES/IFA-vaccinated levels by a square. Non-infected groups are indicated by open symbols while infected groups are indicated by closed symbols. Statistical analyses were carried out from data from the (A) 1:5,120 dilution, from the (B) 1:1,280 dilution and from the (C and D) 1:80 dilution. Significant differences between groups are represented by a * and a table of significant differences between groups is presented in (E). The results presented are from one of two experiments.

Thirty-five days after a primary infection, parasite-specific IgG1 production is associated with a protective Th2 response to *T. muris* in resistant strains of mouse. Conversely, parasite-specific IgG2a production is a feature of the inappropriate Th1 response in susceptible strains of mouse (Else et al., 1993). Thirty-five days after the challenge infection, ES/IFA-vaccinated mice had higher levels of parasite-specific IgG1 in their serum than both untreated naive mice and PBS/IFA-injected control mice (Fig. 5A). At the same time after challenge infection, both the ES/IFA-vaccinated group and the PBS/IFA-injected group produced higher levels of IgG2a than the untreated naive group. Vaccination with ES/IFA resulted in higher levels of IgG2a at 35 days p.i. than injection with PBS/IFA (Fig. 5B).

In order to investigate whether ES/IFA vaccination induced detectable levels of parasite-specific antibodies in the serum during the period of worm expulsion, IgG1 and IgG2a were measured in the serum of mice 10 days post-vaccination but prior to infection (day 0) and again at 21 days post-challenge infection. At day 0, ES/IFA vaccination raised serum IgG1 levels significantly above those in untreated naive mice and in PBS/IFA-injected controls (Fig. 5C). Although there appeared to be even higher levels of IgG1 in ES/IFA-vaccinated mice 21 days after the challenge infection, this was not statistically significant (Fig. 5C). At these time-points, only negligible levels of IgG2a were detected in the serum in any group of mice (Fig. 5D).

### 3.8 Mucosal antibody responses following ES/IFA vaccination

**Fig. 6:**
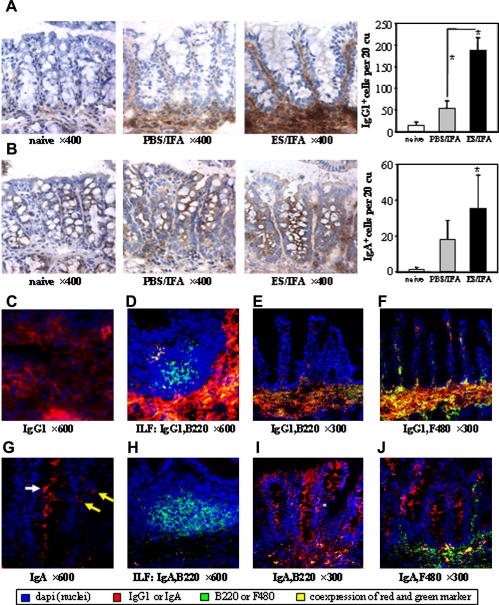
Mucosal antibody responses following ES/IFA vaccination. (A and B) Colorimetric immunohistochemical (IHC) staining and numbers of (A) IgG1^+^ and (B) IgA^+^ (IgA^+^ epithelial and goblet cells not counted) leukocytes in the colonic mucosa of naive and *Trichuris muris*-infected PBS/incomplete Freund’s adjuvant (IFA)-injected and excretory–secretory product (ES)/IFA-vaccinated mice at day 21 p.i. IgG1^+^ and IgA^+^ cells appear brown in colorimetric IHC stained sections. Graphed results are shown as mean cell numbers per 20 crypt units (cu) ± SD (*n *= 5 per group). * above the error bar indicates that cell number is significantly above naive levels (*P *< 0.05). * with a line indicates a significant difference in IgG1^+^ cell numbers (*P *< 0.05) between the infected groups. (C–J) Fluorescent IHC staining of colonic sections from infected ES/IFA-vaccinated mice showing (C) IgG1^+^ (D) ILF B220^+^ and IgG1^+^ (E) B220^+^ and IgG1^+^, (F) F4/80^+^ and IgG1^+^, (G) IgGA^+^ (H) ILF B220^+^ and IgA^+^ (I) B220^+^ and IgA^+^, (J) F4/80^+^ and IgA^+^ cells. (G) White arrow depicts goblet cell staining while yellow arrows depict leukocyte staining for IgA. The results presented are from one of two experiments.

In the ES/IFA-vaccinated mice, 21 days post-challenge infection, strong staining of IgG1 was seen throughout the extracellular matrix of the colonic lamina propria (Fig. 6A). IgG1^+^ cells were present both at the base and along the length of the crypts (Fig. 6A). Negligible IgG1 staining was found in the colonic mucosa of untreated naive mice (Fig. 6A). Following PBS/IFA injection and subsequent challenge infection, only weak extracellular staining of IgG1 was observed in the lamina propria and there were few IgG1^+^ cells present at the base of the crypts (Fig. 6A). Following a challenge infection, there were significantly more IgG1^+^ cells in the colonic lamina propria of ES/IFA-vaccinated mice than PBS/IFA-injected control mice (Fig. 6A). No staining of any sort was seen with the isotype control antibody (data not shown).

In ES/IFA-vaccinated mice, IgA staining was seen at the luminal surface of epithelial cells – especially goblet cells – and on lamina propria leukocytes (LPL) (Fig. 6B). There were significantly more IgA^+^ leukocytes in ES/IFA-vaccinated mice, post-challenge infection, than in untreated naive mice. No staining was seen with the isotype control antibody (data not shown).

Immunofluorescence staining confirmed that the location of IgG1 was both cellular and extracellular (Fig. 6C). The types of cell producing the IgG1 or IgA were revealed by dual immunofluorescence staining. An isolated lymphoid follicle (ILF) is shown in Fig. 6D. A few IgG1^+^ B220^+^ cells were seen near the periphery of the ILF but the majority of B220^+^ ILF cells, in the cortex, do not express IgG1. Both IgG1^+^ and IgG1^−^ B220^+^ cells were encountered in the gut mucosa (Fig. 6E). Most F4/80^+^ cells in the colonic mucosa of ES/IFA-vaccinated mice were IgG1^+^ (Fig. 6F). Immunofluorescence staining revealed that IgA was expressed by both LPL and epithelial cells (Fig. 6G). Although no B220^+^ IgA^+^ cells were found in the ILF (Fig. 6H) they were encountered in the lamina propria (Fig. 6I). Few IgA^+^ F4/80^+^ cells were seen in the gut mucosa; indeed most F4/80^+^ cells were IgA^−^ (Fig. 6J).

## 4 Discussion

In this study, the vaccine conferred rapid and complete protection against *T. muris* to the highly susceptible AKR strain of mouse. With kinetics similar to that observed during natural worm expulsion in the highly resistant BALB/c strain of mouse (Cliffe et al., 2005), the vaccine achieved total expulsion before the parasites reached patency, thereby preventing environmental contamination with eggs. Ultimately, this would be the goal of a human vaccine against *T. trichiura*. While previous studies vaccinated susceptible strains using ES in FCA, protection was slow to develop and rather than sterile immunity, only a reduction in the worm burden was achieved (Else and Wakelin, 1990). Indeed, many anti-nematode vaccine trials in farm animals do not result in sterile immunity (Newton and Munn, 1999). Therefore, our model system may be useful and relevant for investigating both the development of immunity in response to vaccination and the mechanisms of parasite expulsion following challenge infection.

It is not understood how a vaccine administered s.c. may lead to the successful development of immunity against a nematode residing in a distant and distinct site such as the large intestine. Adding to the confusion, tissue-homing effector T cells are thought to be programmed to return to the site in which the antigen was initially encountered and presented (Fabbri et al., 1999). Several potential mechanisms of parenterally induced mucosal immunity have been proposed which, in one way or another, all point to the MLN as the initial site of antigen presentation to T cells. These theories include: the diffusion of antigens through the circulation to the mucosal-associated lymphoid tissue (MALT) (Bouvet et al., 2002), the migration of dendritic cells from the skin to the MALT (Kripke et al., 1990; Enioutina et al., 2000; Belyakov et al., 2004) or the migration of antigen-presenting B cells from the PLN to the MALT (Coffin et al., 1999). The initial site of antigen presentation following s.c. vaccination against *T. muris* has not previously been identified. This study reveals that, soon after s.c. vaccination, it is the PLN cells, but not the MLN cells, which proliferate and release cytokines. Therefore, in our model, the initial antigen presentation seems to occur in the PLN – the local draining lymph nodes of the skin. Further evidence from our laboratory in support of this comes from a different model system in which either PLN cells or MLN cells are adoptively transferred from ES/IFA-vaccinated BALB/c mice into susceptible severe combined immunodeficient (SCID) mice. When the recipient mice are challenged by infection with *T. muris*, PLN cells, but not MLN cells, confer resistance (Dixon and Else, unpublished data).

Various Th2 cytokines, and the Th1 cytokine IFN-γ, are released by PLN cells following vaccination and then by MLN cells following the challenge infection. Notably however, another Th1 cytokine, IL-12, is not produced in response to vaccination or vaccination and challenge infection but is produced by the MLN cells of chronically infected PBS/IFA-injected AKR mice. Therefore, the vaccine fully induces the typical Th2 response seen in primary infections of the naturally resistant BALB/c mouse but only partially induces the classical Th1 response seen in primary infections of the naturally susceptible AKR mouse. IL-12 is important for initial polarisation towards a Th1 response (O’Garra and Murphy, 2009) and absence of this cytokine may be critical to the development of a protective immune response to *T. muris*. While the vaccine does elicit a mixed Th1/Th2 response with both the Th2-associated IgG1 and the Th1-associated IgG2a produced at day 35 post-challenge infection, only the Th2-associated IgG1 is produced during the critical period for worm expulsion (day 0–21 post-challenge). Thus, the present study suggests that similar to primary infection a Th2 response – regardless of a concomitant or developing Th1 response – mediates the expulsion of *T. muris* in a secondary infection.

In a primary infection, the migration of T helper cells to the large intestine is critically required for the expulsion of *T. muris* (Picarella et al., 1997; Betts et al., 2000) but colonic CD4^+^ cell accumulation can be demonstrated in both resistant and susceptible strains. To clarify, such migration of leukocytes in susceptible AKR mice is the result of the inappropriate Th1 response mounted by this strain to the parasite. Similarly, there is an accumulation of CD4^+^ cells in the gut following a challenge infection in both ES/IFA-vaccinated and PBS/IFA-injected control mice. Recently, several locally acting T-cell-dependent effector mechanisms have been elucidated which are ultimately responsible for the expulsion of *T. muris* in a primary infection. The effector mechanisms underlying the expulsion of worms from vaccinated mice challenged by a *T. muris* infection have not been previously investigated.

In resistant BALB/c mice, an increase in the rate of epithelial turnover has been shown to be a key effector mechanism in the expulsion of worms (Cliffe et al., 2005). This IL-13-dependent event results in the shortening of epithelial crypts. In contrast, during a chronic infection in susceptible AKR mice, IFN-γ-driven intestinal epithelial cell hyperproliferation occurs (Artis et al., 1999; Cliffe et al., 2005). This is responsible for crypt hyperplasia, leading to an increase in the length of the epithelial crypts. Hence, resistant BALB/c mice have shorter crypts than susceptible AKR mice during a primary infection (Cliffe et al., 2005). In the present study, both ES/IFA-vaccinated and PBS/IFA-injected challenged mice had longer crypts than naive mice, suggesting that crypt hyperplasia occurs during the vaccine-induced protective immune response to *T. muris*. This is probably brought about by the high levels of IFN-γ which accompany the response. Hence, although an increase in the rate of epithelial cell turnover results in worm expulsion in naturally resistant BALB/c mice during a primary infection (Cliffe et al., 2005), this mechanism would not appear to be implicated in the expulsion of worms by vaccinated mice during a challenge infection.

Despite the marked eosinophilia and mastocytosis in the gut during a primary infection, worm expulsion can proceed unhindered in the absence of eosinophils and mast cells (Betts and Else, 1999; Koyama and Ito, 2000; Dixon et al., 2006). Eosinophils are able to kill the larvae of migratory nematodes such as *Trichinella spiralis* (Grove et al., 1977; Wassom and Gleich, 1979; Gurish et al., 2002) and *Brugia malayi* (Simons et al., 2005). Mast cells are required for the expulsion of the small intestinal nematode *T. spiralis* (Grencis et al., 1993). Therefore, in our vaccination and challenge model, the greater influx of eosinophils and mast cells following ES/IFA vaccination than PBS/IFA injection may merely indicate a general type 2 anti-nematodal response in the former. However, eosinophils may contribute to ADCC involving IgA or IgG1 (bu-Ghazaleh et al., 1989; Khalife et al., 1989; Nutten et al., 1999) as eosinophils mediate ADCC against helminths in vitro (Butterworth et al., 1975; Glauert and Butterworth, 1977; Venturiello et al., 1995). To address the role for eosinophils in vaccination-induced protection, vaccination of an eosinophil-deficient mouse model, such as the Δdbl GATA-1 mouse (Yu et al., 2002) would be required. Although mast cells are not thought to be important in the mechanisms of resistance to a primary infection (Betts and Else, 1999; Koyama and Ito, 2000) their local elevation in vaccinated infected mice may indicate a role in protection after vaccination. Vaccination of mice depleted of mast cells using anti-stem cell factor antibodies or transgenic mice deficient in mucosal mast cell protease-1 (Knight et al., 2000) will allow the role of the mast cell and its products in vaccine-induced protection to be critically assessed.

The role macrophages play in the mechanism of resistance to *T. muris* is still unclear in both primary and challenge infections. CCL2-deficient mice exhibit reduced recruitment of macrophages to the colonic mucosa and are also unable to expel *T. muris* during a primary infection (Deschoolmeester et al., 2003). Alternatively activated macrophages have been implicated in the expulsion of a variety of parasites, including several nematodes (Anthony et al., 2006). However, the accumulation of alternatively activated macrophages in both ‘naturally resistant’ BALB/c and ‘vaccinated resistant’ AKR mice at day 21 p.i., a time-point at which most of the worms have already been expelled from the gut, may reflect the role of the macrophage in the resolution of inflammation and in directing the repair of damaged tissue (Shearer et al., 1997; Liu et al., 2004).

In the present study, colonic goblet cell hyperplasia, in response to the challenge infection, occurs only in the vaccinated mice. Goblet cell hyperplasia, which is IL-13-dependent (McKenzie et al., 1998), is also a prominent response to a primary infection in resistant strains of mouse (Datta et al., 2005; Owyang et al., 2006). The release of RELM-β by goblet cells during infection is thought to inhibit the chemotaxis of *T. muris* into the epithelium, thereby dislodging the parasite into the lumen (Artis et al., 2004). This putative mechanism of expulsion may also play a role in the vaccine-driven response to a challenge infection.

Following the primary infection of resistant strains of mouse with *T. muris* there is an appreciable increase in the levels of parasite-specific IgG1 in the serum. However, this develops after the parasite has already been expelled (Else et al., 1993). Therefore, rather than playing a role in the expulsion of a primary infection, IgG1 is more likely to be involved in the memory response of the adaptive immune system to a subsequent infection. Significantly, early studies showed that while immunity could be transferred with either the serum or cells of protected mice, the highest levels of protection were achieved by the transfer of serum (Selby and Wakelin, 1973). Furthermore, the adoptive transfer of purified *T. muris*-specific IgG1 provides partial protection to susceptible strains of mouse (Blackwell and Else, 2001) while μMT mice, which lack B cells, cannot be protected against *T. muris* by s.c. vaccination (Blackwell and Else, unpublished data). In the present study, parasite-specific IgG1 is present at high levels in the serum following vaccination prior to infection and at day 21 post-challenge infection towards the end of worm expulsion. Therefore, IgG1-dependent mechanisms may be central to the expulsion of *T. muris* in vaccinated mice. Since *T. muris* resides in the large intestine, in order to be effective IgG1 must be present in the colonic mucosa. Indeed, we show IgG1 located in the extracellular matrix of the lamina propria and significantly increased numbers of IgG1^+^ B cells and macrophages in the colonic mucosa of vaccinated mice following the challenge infection. Future studies will analyse local tissue levels of IgG1 at early time-points post-infection in order to correlate this with larval clearance. IgA is also present in the colonic mucosa of vaccinated resistant AKR mice and therefore it may also be involved in the expulsion mechanism. The adoptive transfer of *T. muris*-specific monoclonal IgA antibodies has previously been shown to reduce the worm burden (Roach et al., 1991) and IgA has previously been shown in the colon following mucosal vaccination, correlating with resistance to *T. muris* (Robinson et al., 1995). IgA is secreted into the lumen of the intestine and by binding to molecules on the nematode surface it could prevent nematode adhesion to the colon or facilitate worm killing by ADCC (Nutten et al., 1999).

This paper describes a model system of s.c. vaccination against *T. muris* in which sterile immunity is conferred to susceptible mice. To our knowledge for the first time, the initial immune response to such a vaccine was investigated and was shown to occur in the PLN rather than the MLN. This is also the first study to describe the recruitment of leukocytes into the large intestine in vaccinated mice following a challenge infection. Although the expulsion mechanisms operating in each model are yet to be fully elucidated, it seems that while accelerated epithelial turnover is essential in the expulsion of a primary infection, it is unlikely to play a role in the mechanism of vaccine-induced resistance. Potentially, antibody-dependent mechanisms play a more prominent role in the vaccine-induced expulsion of worms. Using this vaccination model, future work in our laboratory will establish which elements of the immune response are required to confer protection against *T. muris*. Through long-term studies it will also be important to identify the longevity of protection offered by vaccination and to establish vaccination regimes which maximise the duration of action. This will further aid the rational design of an anti-*Trichuris* vaccine. Since all currently licensed vaccines work by eliciting an antibody response directed at the pathogen, the involvement of antibodies in vaccine-induced protection against *T. muris* would be encouraging.
